# Integrated microbiology and metabolomic analysis reveal the improvement of rice straw silage quality by inoculation of *Lactobacillus brevis*

**DOI:** 10.1186/s13068-023-02431-y

**Published:** 2023-11-28

**Authors:** Yu Sun, Qinglong Sun, Yunmeng Tang, Qingyang Li, Chunjie Tian, Haixia Sun

**Affiliations:** 1 State Key Laboratory of Black Soils Conservation and Utilization, Northeast Institute of Geography and Agroecology, Chinese Academy of Sciences, Changchun, 130102 China; 2 Key Laboratory of Mollisols Agroecology, Northeast Institute of Geography and Agroecology, Chinese Academy of Sciences, Changchun, 130102 China; 3 Northeast Institute of Geography and Agroecology, Chinese Academy of Sciences, Harbin, 150081 China; 4 Northeast Agricultural University, Harbin, 150030 China

**Keywords:** Lactic acid bacteria, Rice straw silage, Fermentation quality, Bacterial community, Metabolite profiles

## Abstract

**Background:**

Ensiling technology holds promise for preserving and providing high-quality forage. However, the preservation of rice straw poses challenges due to its high lignocellulosic content and low water-soluble carbohydrate levels. Developing highly effective lactic acid bacteria (LAB) for rice straw silage remains a priority.

**Results:**

This study evaluated the impact of three LAB strains, *Lactobacillus brevis* R33 (Lac33), *L. buchneri* R17 (Lac17), and *Leuconostoc pseudomesenteroides* (Leu), on the fermentation quality of rice straw silage. Rice straw silage inoculated with Lac33 alone or in combination with other strains exhibited significantly lower neutral detergent fiber (NDF) (66.5% vs. 72.3%) and acid detergent fiber (ADF) (42.1% vs. 47%) contents, along with higher lactic acid (19.4 g/kg vs. not detected) and propionic acid (2.09 g/kg vs. 1.54 g/kg) contents compared to control silage. Bacterial community analysis revealed *Lactobacillus* dominance (> 80%) and suppression of unwanted *Enterobacter* and *Clostridium*. Metabolomic analysis highlighted increased carbohydrates and essential amino acids, indicating improved nutrient values in Lac33-inoculated rice straw silage and a potential explanation for Lac33 dominance.

**Conclusions:**

This research identified a highly efficient LAB candidate for rice straw silage, advancing our comprehension of fermentation from integrated microbiology and metabolomic perspectives.

**Supplementary Information:**

The online version contains supplementary material available at 10.1186/s13068-023-02431-y.

## Introduction

Rice, a staple crop that feeds over 50% of the global population, generates a substantial amount of rice straw, which is often ineffectively disposed of, leading to environmental pollution [1, 2]. Utilizing rice straw in livestock production can mitigate this issue and alleviate feed scarcity. However, its high fiber, lignin, and silica content, along with low water-soluble carbohydrate levels, hinder digestion and reduce its nutritive value for ruminants [3].

Various treatments have been attempted to enhance the digestibility of rice straw, including physical and chemical methods [4]. Nevertheless, the drawbacks of these treatments limit their widespread application [5]. Ensiling, a preservation technique that yields high-quality forage for ruminants, offers a potential solution [6]. Ensilage retains more than 90% of plant energy and improves the efficiency of enzymatic hydrolysis when compared to raw material [7].

Lactic acid bacteria (LAB), known for their ability to lower pH and inhibit undesirable microorganisms, have been extensively utilized to enhance the fermentation process and improve the nutrient value of rice straw silage [8]. LAB strains associated with silage predominantly belong to the bacterial genera *Lactobacillus*, *Enterococcus*, and *Pediococcus* [4]. For example, *L. plantarum*, a homofermentative strain producing lactic acid rapidly, has been shown to increase in vitro dry matter digestibility (IVDMD) while having no effect on neutral detergent fiber (NDF) and acid detergent fiber (ADF) contents of rice straw silage [9]. Due to the challenge of ensiling rice straw with LAB, and the potential proliferation of undesirable microorganisms owing to its high lignocellulosic content and low water-soluble carbohydrate (WSC) content [10, 11], researchers have explored the addition of high WSC products such as molasses and other high-moisture feed to improve LAB colonization efficiency [11–13]. Additional strategies, including the addition of cellulase in conjunction with LAB or the creation of genetically engineered LAB strains with cellulase genes, have also been investigated [14, 15]. Given the current state of research on rice straw silage, the development of highly effective LAB capable of thriving in low WSC conditions and reducing NDF and ADF contents in rice straw silage remains an ongoing challenge. Furthermore, previous studies have primarily focused on traditional chemical indicators of silage, such as NDF, ADF, and specific acids, while comprehensive and detailed characterization of rice straw silage using metabolomic analysis has been limited.

In this study, two LAB strains (*L. brevis* R33 and *Leuconostoc pseudomesenteroides*) that exhibit cellulase-producing capabilities were isolated from oats silage. The effects of these strains and another previously isolated LAB strain (*L. buchneri* R17) on the chemical composition, bacterial communities, and metabolite profiles of rice straw silage were investigated. This study not only identifies a new and potentially effective LAB candidate for rice straw silage but also offers new insights into the role of LAB in enhancing the quality of rice straw silage, from both microbiological and metabolomic perspectives.

## Results and discussion

### Chemical composition and fermentation quality of rice straw silage with inoculation of different strains

**Fig. 1:**
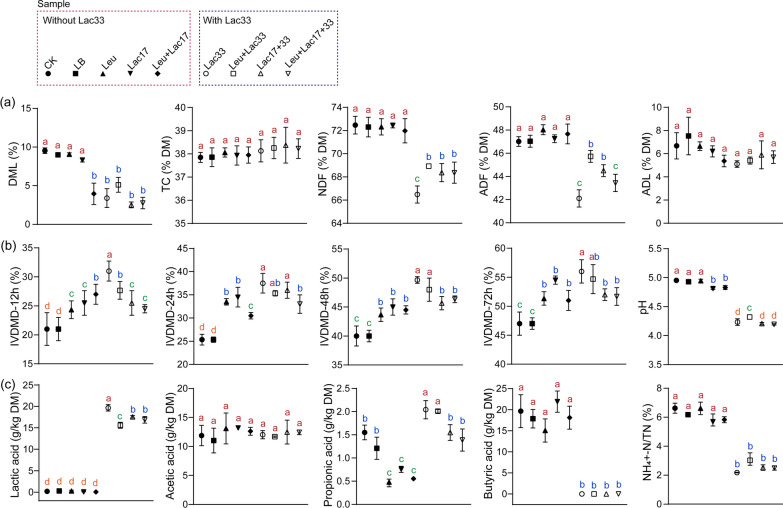
Chemical characteristics of rice straw silage with different strain inoculations. The symbols and error bars represent the mean value and standard deviation of six biological replicates, respectively. Distinct letters on top indicate significant differences across all combinations (Tukey’s honestly significant difference test, *P* < 0.05). Dashed rectangles in red and blue depict groups inoculated without Lac33 and with Lac33, respectively. Abbreviations: CK, no inoculation; LB, inoculated with LB; Leu, inoculated with *L. pseudomesenteroides*; Lac17, inoculated with *L. buchneri* R17; Leu + Lac17, inoculated with *L. pseudomesenteroides* and *L. buchneri* R17; Lac33, inoculated with *L. brevis* R33; Leu + Lac33, inoculated with *L. pseudomesenteroides* and *L. brevis* R33; Lac17 + 33, inoculated with *L. buchneri* R17 and *L. brevis* R33; Leu + Lac17 + 33, inoculated with *L. pseudomesenteroides*, *L. buchneri* R17, and *L. brevis* R33. DML, dry matter loss; NDF, neutral detergent fiber; ADF, acid detergent fiber; ADL, acid detergent lignin; IVDMD, in vitro dry matter digestibility

To discern the influence of different strains on the chemical composition of rice straw silage, an initial assessment of its composition was conducted. Notably, samples inoculated solely with Leu or Lac17 demonstrated comparable Dry Matter Loss (DML) to control treatments, whereas samples receiving a combined inoculation of Leu and Lac17 exhibited lower DML than the control (Fig. 1a). Remarkably, inoculation with Lac33, whether alone or in combination with other strains, led to a significant reduction in DML, nearly tripling the efficacy (Fig. 1a). Conversely, neither Total Carbon (TC) nor crude protein content was affected by strain inoculation (Fig. 1a and Additional file 1: Fig. S1). Whereas inoculation with Leu, Lac17, and their combination had no discernible impact on Neutral Detergent Fiber (NDF) and Acid Detergent Fiber (ADF) contents, inoculation with Lac33 yielded a substantial decrease in both NDF (from 72.3% to 66.5%) and ADF (from 47% to 42.1%) contents (Fig. 1a). Moreover, Lac33 inoculation exhibited a marginal but non-significant reduction in Acid Detergent Lignin (ADL) content (Fig. 1a). The improved fiber content resulting from Lac33 inoculation can be attributed to the potential degradation of crude fiber facilitated by enzyme activities such as endoglucanase, exoglucanase, and β-glucosidase associated with Lac33 (Additional file 1: Fig. S2). Lac33 inoculation also led to a significant enhancement in In Vitro Dry Matter Digestibility (IVDMD), particularly over 72 h (Fig. 1b). However, co-inoculation with other strains seemed to marginally attenuate the efficiency of Lac33. Given the recalcitrance of lignocellulosic structure, the reduction of fiber content in silage can alleviate intake and digestibility limitations in livestock [16], thus contributing to an improved nutritional value.

The pH values of the silage decreased from 4.95 to 4.81 and 4.83 with the inoculation of Lac17 and the combination of Lac17 and Leu, respectively. In contrast, Lac33 inoculation led to a significant pH reduction from 4.95 to 4.2 (Fig. 1b). In parallel with the lowered pH, Lac33 inoculation substantially elevated lactic acid content from undetectable levels to 19.4 g/kg, while also increasing propionic acid content from 1.54 g/kg to 2.09 g/kg (Fig. 1c). The acetic acid content remained unaffected by strain inoculation (Fig. 1c). The presence of high levels of lactic and propionic acids can effectively lower pH and deter undesired bacterial growth during fermentation [8, 17]. Furthermore, Lac33 inoculation resulted in a notable decrease in butyric acid content from 19.7 g/kg to undetectable levels (Fig. 1c). Butyric acid, produced primarily by Clostridia, can manifest in under-fermented silage, signifying dry matter loss, energy inefficiency, and potential livestock health concerns [18]. Lac33's suppression of butyric acid production can be attributed to the inhibition of Clostridia. The high levels of ammonia-N in silage often indicate excessive protein degradation during fermentation [19]. However, the ratio of NH_4_^+^-N/TN was significantly reduced from 6.6% to 2.2% by Lac33 inoculation alone or in combination with other strains (Fig. 1c). This decrease can be attributed to the rapid pH decline that hampers specific microbial growth and plant enzyme activity, subsequently curbing protein degradation [6]. In summary, the elevation of lactic and propionic acids coupled with the reduction of butyric acid and ammonia-N collectively contribute to the heightened quality of rice straw silage with Lac33 inoculation.

### Effects of inoculation of different strains on the bacterial communities of rice straw silage

**Fig. 2:**
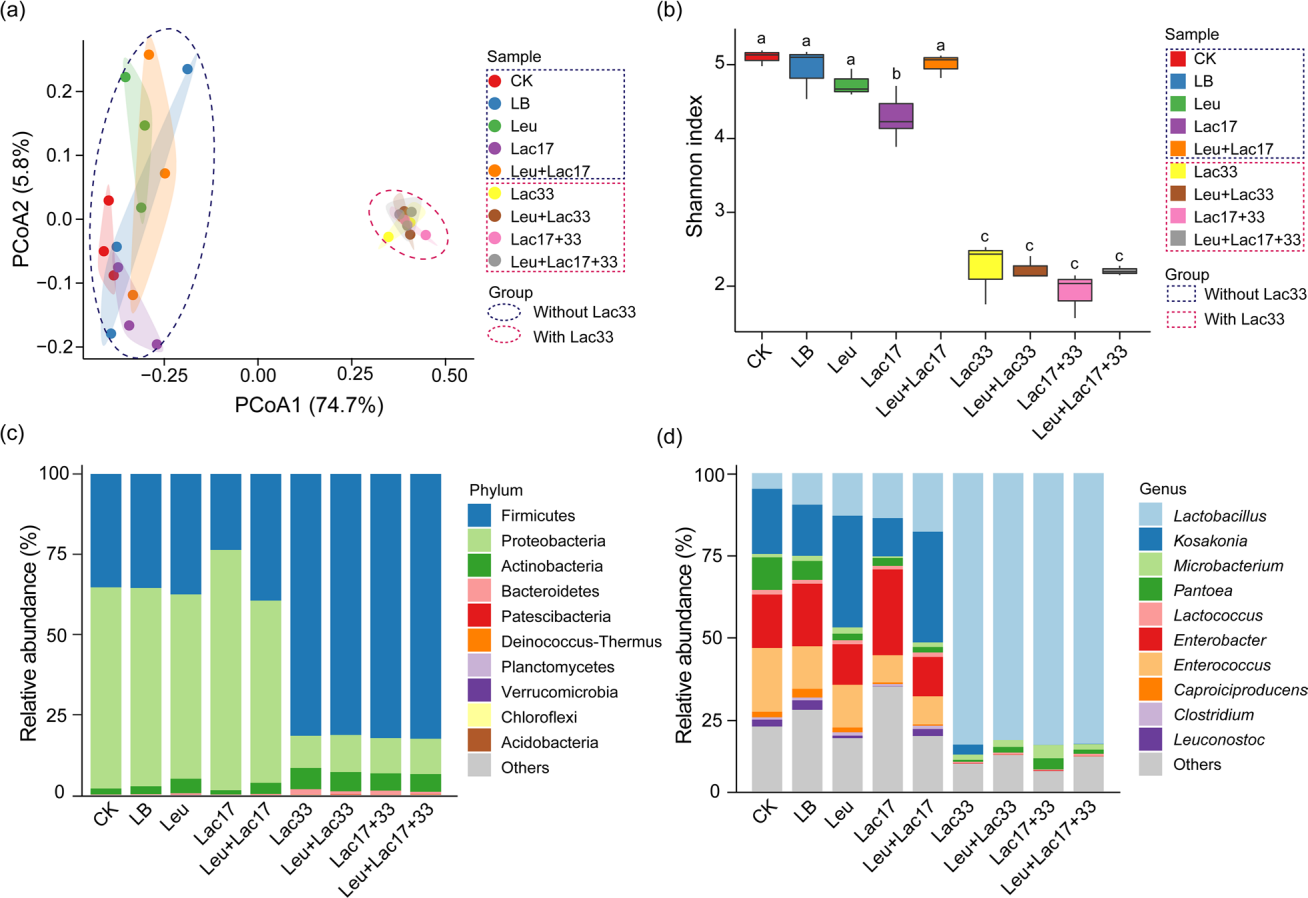
Analysis of bacterial communities in rice straw silage with various strain inoculations. **a** Principal coordinate analysis (PCoA) of bacterial communities in rice straw silage inoculated with different strains. The 95% confidence ellipses are depicted in respective colors for diverse communities. Dashed circles in blue and red signify groups inoculated without Lac33 and with Lac33, respectively. **b** Shannon indices of different samples. Boxplots indicate median, 25th and 75th percentiles, while error bars represent standard deviations of three replicates. **c** Relative abundance of phylum and **d** genus in bacterial communities of distinct silage samples. CK, no inoculation; LB, inoculated with LB; Leu, inoculated with *L. pseudomesenteroides*; Lac17, inoculated with *L. buchneri* R17; Leu + Lac17, inoculated with *L. pseudomesenteroides* and *L. buchneri* R17; Lac33, inoculated with *L. brevis* R33; Leu + Lac33, inoculated with *L. pseudomesenteroides* and *L. brevis* R33; Lac17 + 33, inoculated with *L. buchneri* R17 and *L. brevis* R33; Leu + Lac17 + 33, inoculated with *L. pseudomesenteroides*, *L. buchneri* R17, and *L. brevis* R33

To probe the influence of distinct strain inoculations on rice straw silage bacterial communities, we conducted principal coordinate analysis (PCoA) on the bacterial compositions of silage treated with various strains. The PCoA plots unveiled discernible differences between the treatments, wherein the rice silage samples were distinctly partitioned along the first principal coordinate into two groups: with and without Lac33 (Fig. 2a and Additional file 1: Table S1). This disparity indicated that the introduction of Lac33 significantly altered the bacterial community compositions within the rice silage. Furthermore, the Shannon indices of rice silage samples with Lac33 exhibited a notable reduction compared to those without Lac33 (Fig. 2b). This phenomenon could be attributed to the decreased pH levels that could potentially impede the growth of diverse bacterial species [20].

The taxonomic compositions of silage bacterial communities were subsequently explored at both the phylum and genus levels. In the absence of Lac33 inoculation, Proteobacteria stood as the most abundant phylum, constituting over 60% of the entire community. Conversely, Lac33 inoculation induced a dominance of Firmicutes, accounting for over 80% of the community (Fig. 2c). Additionally, the relative abundances of Actinobacteria and Bacteroidetes were higher in samples with Lac33 than those without (Fig. 2c). Notably, the restraint of Proteobacteria, known for its capability to degrade organic matter in anaerobic environments, by Lac33 inoculation might elucidate the diminished dry matter (DM) loss during fermentation [21]. Further granularity emerged from the genus-level distribution of bacteria, yielding insights into community changes. Silage samples lacking Lac33 exhibited elevated bacterial diversity, mirroring the higher Shannon indices observed (Fig. 2b). The top five dominant genera in these samples were *Kosakonia* (11.5–34.5%), *Enterobacter* (12.3–26.9%), *Enterococcus* (8.6–20.1%), *Lactobacillus* (4.9–18.4%), and *Pantoea* (1.7–10.2%) (Fig. 2d). In stark contrast, *Lactobacillus* dominated the entirety of the bacterial communities in silage samples with Lac33 (83.9%-85.3%) (Fig. 2d). Well-established as a representative of lactic acid bacteria (LAB), *Lactobacillus* assumes a pivotal role in generating substantial lactic acid quantities and reducing pH levels, consequently establishing dominance in higher-quality silages [22].

**Fig. 3:**
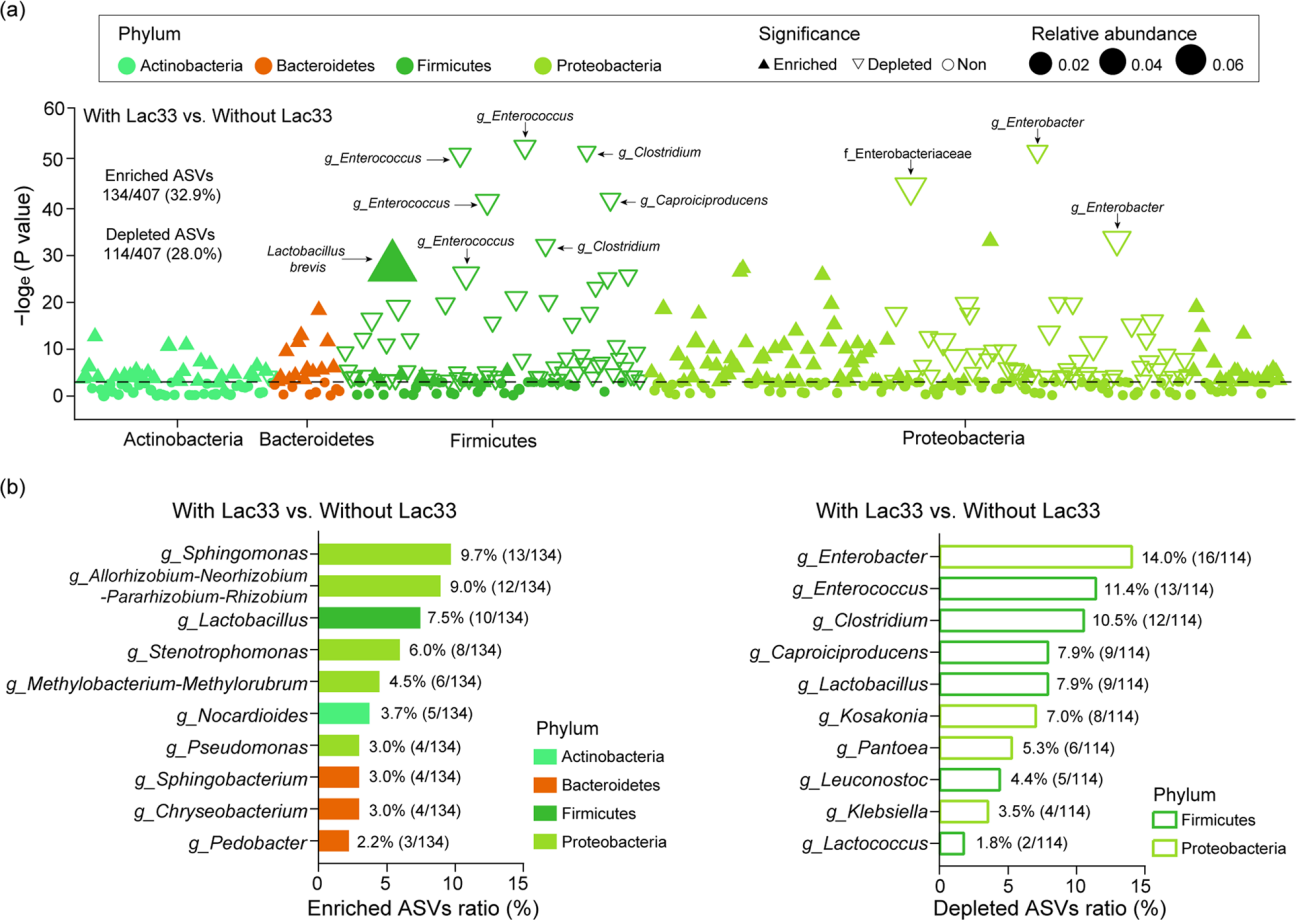
Taxonomic characterization of differential bacterial taxa between silage groups with and without L. brevis R33 inoculation. **a** Manhattan plots display ASVs enriched or depleted between bacterial communities of silage groups with and without *L. brevis* R33 inoculation. Specific ASVs are labeled with corresponding species, genus, or family. Filled triangles represent enriched ASVs, empty triangles signify depleted ASVs, and filled circles denote non-significant comparisons (FDR-adjusted *P* < 0.05, Wilcoxon rank sum test). Triangle and circle sizes are proportionate to ASV relative abundances. **b** Analysis of enriched and depleted ASVs summarized and displayed at the genus level. Abbreviations: CK, no inoculation; LB, inoculated with LB; Leu, inoculated with *L. pseudomesenteroides*; Lac17, inoculated with *L. buchneri* R17; Leu + Lac17, inoculated with *L. pseudomesenteroides* and *L. buchneri* R17; Lac33, inoculated with *L. brevis* R33; Leu + Lac33, inoculated with *L. pseudomesenteroides* and *L. brevis* R33; Lac17 + 33, inoculated with *L. buchneri* R17 and *L. brevis* R33; Leu + Lac17 + 33, inoculated with *L. pseudomesenteroides*, *L. buchneri* R17, and *L. brevis* R33

To comprehensively assess the dynamics of enrichment and depletion of specific bacteria, we employed a Manhattan plot approach. Examination of these plots revealed that ASVs that were enriched (133 ASVs) or depleted (114 ASVs) in the comparison between Lac33-inoculated and non-inoculated samples predominantly belonged to four phyla: Actinobacteria, Bacteroidetes, Firmicutes, and Proteobacteria (Fig. 3a). Most notably, the ASV that exhibited the most substantial enrichment in Lac33-inoculated samples was identified as *Lactobacillus brevis*, underscoring the successful colonization and thriving of the introduced *L. brevis* R33 throughout fermentation, culminating in its dominance within the final bacterial communities of the silage (Fig. 3a). A concise overview of enriched ASVs unveiled that *Sphingomonas* (13 ASVs), *Allorhizobium-Neorhizobium-Pararhizobium-Rhizobium* (12 ASVs), *Lactobacillus* (10 ASVs), and *Stenotrophomonas* (8 ASVs) emerged as the most enriched genera within Lac33-inoculated samples. Earlier studies have reported the ascendancy of *Stenotrophomonas* in mixed silage composed of alfalfa, rice straw, and wheat bran following 45 days of fermentation [23], while *Sphingomonas* has been identified as a favored genus in sweet sorghum silage [24]. Notably, the increased presence of *Lactobacillus* aligns with its pivotal role within the final bacterial communities of silage samples treated with Lac33.

Furthermore, examination of significantly depleted ASVs in Lac33-inoculated samples revealed associations with the genera *Caproiciproducens*, *Clostridium*, and *Enterococcus*-phylum Firmicutes, and *Enterobacter* and Enterobacteriaceae (at the family level)-phylum Proteobacteria (Fig. 3a). The summary of depleted ASVs underscored that *Enterobacter* (16 ASVs), *Enterococcus* (13 ASVs), *Clostridium* (12 ASVs), and *Caproiciproducens* (9 ASVs) were the most significantly depleted genera (Fig. 3b). *Enterococcus*, recognized as one of the typical LAB [4] (Oladosu et al.), has been reported by Bai et al. [25] to exhibit reduced abundance upon *Lactobacillus* inoculation. In our study, the decline in *Enterococcus* could potentially stem from competition with *Lactobacillus*. It is worthwhile noting that *Caproiciproducens* and *Clostridium*, which are capable of producing carbon dioxide and butyric acid while competing with *Lactobacillus* under anaerobic conditions, have been associated with compromised fermentation quality and reduced feeding value in silage [26, 27]. Their depletion may also account for the complete inhibition of butyric acid production in Lac33-inoculated samples (Fig. 1c). *Enterobacter*, recognized for deaminating and decarboxylating certain amino acids, thereby producing ammonia and biogenic amines that reduce nutritional value and palatability of silage [5, 18], is widely considered an undesirable microbe in silage [28]. The decrease in *Enterobacter* abundance aligns with the diminished deamination activity, represented by the lowered ammonia-N content in Lac33-inoculated samples (Fig. 1c). The prompt pH decrease, inhibiting the growth of these detrimental microbes [4], is likely attributable to the lactic acid produced by *L. brevis* R33, potentially driving the reduction in their abundance. In summary, the inoculation of *L. brevis* R33 serves as a significant contributor to the enhancement of fermentation quality in rice straw silage, as evidenced by the enrichment of beneficial microbial populations and the reduction of undesirable ones.

### Effects of inoculation of different strains on the metabolite profiles of rice straw silage

**Fig. 4:**
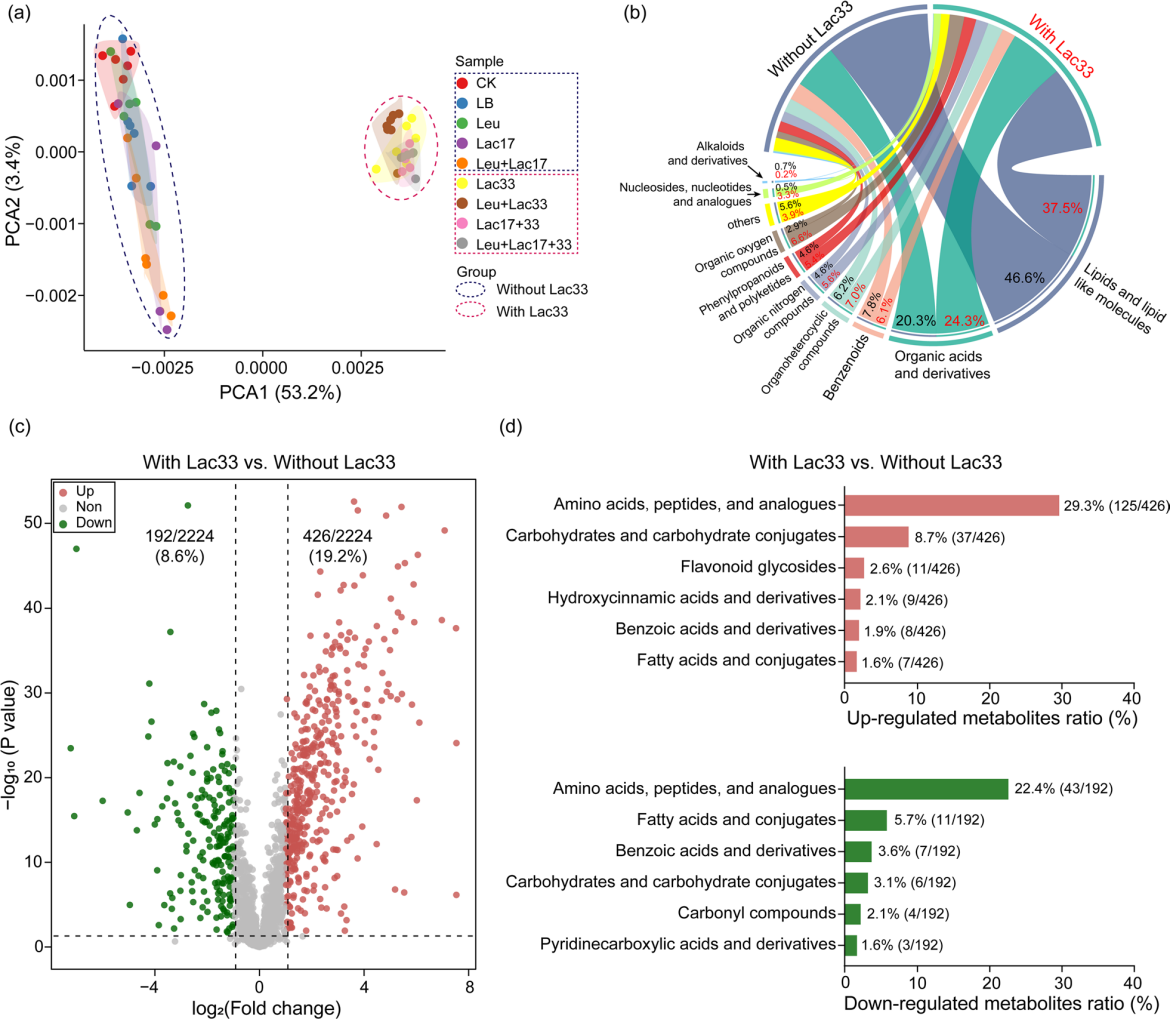
Metabolite analysis of rice straw silage with various strain inoculations. **a** Principal Component Analysis (PCA) of metabolite profiles in rice straw silage with different strain inoculations. The 95% confidence ellipses are shown in respective colors for distinct communities. Dashed circles in blue and red signify groups inoculated without Lac33 and with Lac33, respectively. **b** Relative abundance of metabolite categories in silage groups with and without *L. brevis* R33 inoculation. Metabolites classified into different groups and labeled accordingly. **c** Volcano plots illustrate metabolites up or down-regulated between silage groups without and with *L. brevis* R33 inoculation. Dashed lines represent fold change (FC > 2 and < -2) and *P-*value (*P* < 0.05) thresholds. **d** Analysis of up and downregulated metabolites summarized and displayed at the subclass level. Abbreviations: CK, no inoculation; LB, inoculated with LB; Leu, inoculated with *L. pseudomesenteroides*; Lac17, inoculated with *L. buchneri* R17; Leu + Lac17, inoculated with *L. pseudomesenteroides* and *L. buchneri* R17; Lac33, inoculated with *L. brevis* R33; Leu + Lac33, inoculated with *L. pseudomesenteroides* and *L. brevis* R33; Lac17 + 33, inoculated with *L. buchneri* R17 and *L. brevis* R33; Leu + Lac17 + 33, inoculated with *L. pseudomesenteroides*, *L. buchneri* R17, and *L. brevis* R33

To delve deeper into the metabolic consequences of different strain inoculations, we conducted a metabolomic analysis of rice straw silage. Given the observed shifts in bacterial communities due to Lac33 inoculation, we anticipated concurrent changes in the metabolite profiles. As expected, Principal Component Analysis (PCA) of the metabolite profiles distinctly grouped Lac33-inoculated and non-inoculated samples, indicating distinct metabolite profiles due to Lac33 inoculation (Fig. 4a and Additional file 1: Table S2). A total of 2224 metabolites were identified, predominantly spanning 10 categories, and their relative abundances diverged between the two groups (Fig. 4b and Additional file 2). Notably, “lipids and lipid-like molecules” and “organic acids and derivatives” emerged as the most abundant categories, collectively constituting over 60% of the total metabolite abundance. Intriguingly, samples lacking Lac33 exhibited higher abundance of “lipids and lipid-like molecules” (46.6%) compared to samples with Lac33 (37.5%). In contrast, “organic acids and derivatives” were more abundant in Lac33-treated samples (24.3%) compared to non-Lac33 samples (20.3%) (Fig. 4b). Additionally, other categories such as “organoheterocyclic compounds”, “organic nitrogen compounds”, “phenylpropanoids and polyketides”, “organic oxygen compounds”, and “nucleosides, nucleotides, and analogues” also exhibited higher abundance in Lac33-inoculated samples (Fig. 4b).

To comprehend the impact of Lac33 inoculation, we examined up and downregulated metabolites through Volcano plot analysis. This analysis revealed that among the 2224 metabolites, 426 (19.2%) were upregulated, and 192 (8.6%) were down-regulated upon Lac33 inoculation. The upregulated metabolites were primarily associated with the subcategories of “amino acids, peptides, and analogues” (29.3%, 125/426), “carbohydrates and carbohydrate conjugates” (9.7%, 37/426), and “flavonoid glycosides” (2.6%, 11/426) (Fig. 4d). The increased abundance of amino acids and carbohydrates, stemming from the breakdown of complex molecules such as cellulose, hemicellulose, and lignin, could potentially offer additional nitrogen and carbon resources to fermenting microbes, notably *Lactobacillus*, enhancing lactic acid production [29, 30]. Moreover, higher amino acid and carbohydrate content in rice straw silage can render it more digestible for ruminant animals [31, 32]. Intriguingly, the abundance of ferulic acid, released through the hydrolysis of lignin-linking bonds by ferulic acid esterase [33], was markedly elevated in Lac33-inoculated silage samples, potentially augmenting silage degradability and nutritive value (Additional file 1: Fig. S3). This elevation might be indicative of increased ferulic acid esterase activity, which merits further investigation. The augmented abundance of flavonoids, known for their anti-biohydrogenation and antioxidant properties in silage [34], further suggests their contribution to enhanced silage quality. On the flip side, certain metabolites from subcategories such as “amino acids, peptides, and analogues” (22.4%, 43/192) and “fatty acids and conjugates” (5.7%, 11/192) were downregulated in Lac33-inoculated samples (Fig. 4d). This could be attributed to the dominance of *Lactobacillus*, which could inhibit the activity of other microbes such as *Clostridium*, thus impacting specific fatty acid synthesis, including butyric acid [12].

**Fig. 5:**
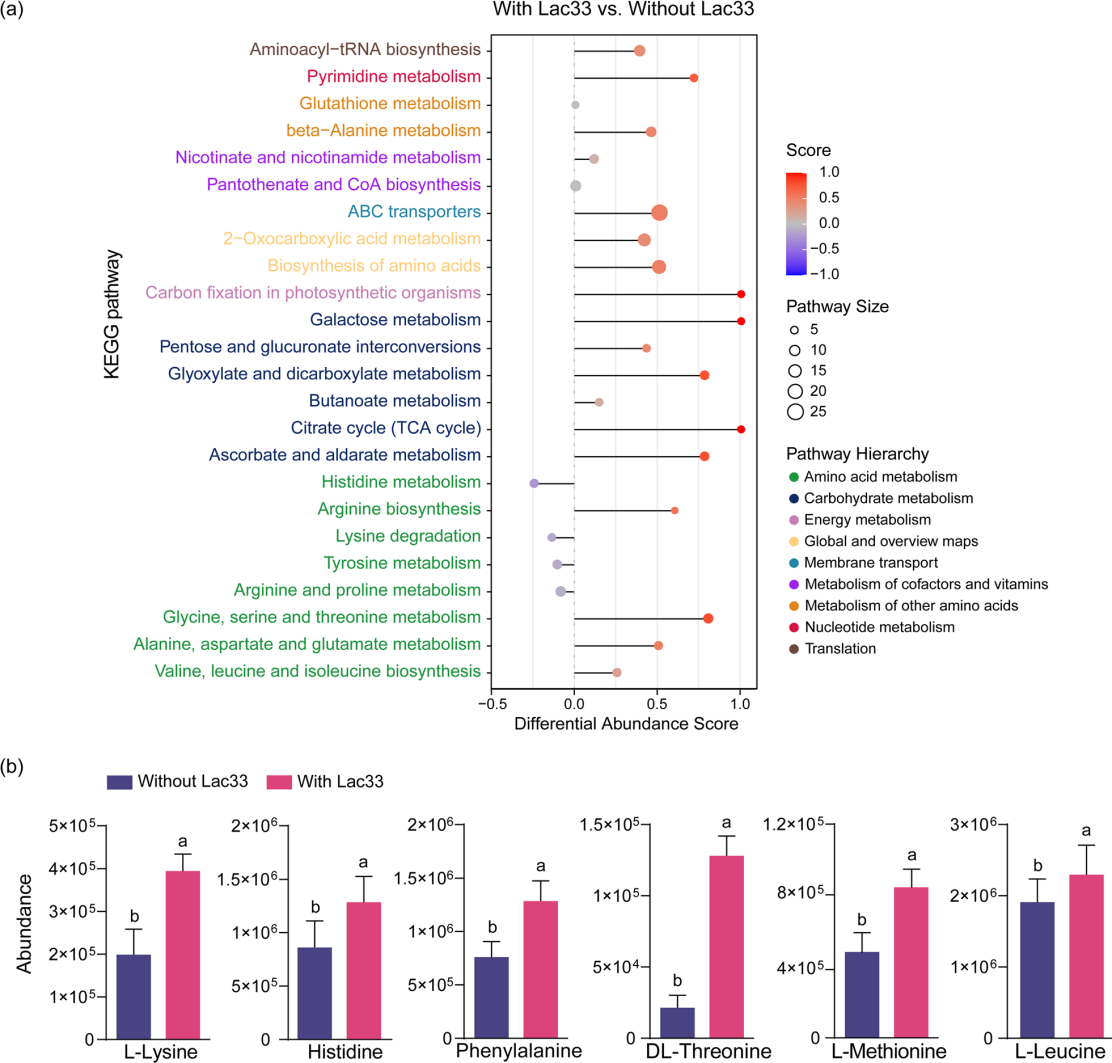
KEGG pathway enrichment analysis of differential metabolites between silage groups with and without *L. brevis* R33 inoculation. **a** Differential abundance scores of KEGG pathways between silage groups with and without *L. brevis* R33 inoculation. Pathway size corresponds to differential metabolite counts. Differential abundance scores: 1 signifies upregulated differential metabolites; -1 represents downregulated differential metabolites. **b** Abundance of specific amino acids in silage groups with and without *L. brevis* R33 inoculation. Columns and error bars denote mean value and standard deviation of biological replicates (Without R33, *n* = 30; With R33, *n* = 24), respectively. Different letters on top indicate significant differences (Tukey’s honestly significant difference test, *P* < 0.05)

To elucidate the specific pathways affected by *L. brevis* R33 inoculation, we conducted a KEGG pathway enrichment analysis of metabolites. Results revealed the involvement of diverse pathways, encompassing “amino acid metabolism”, “carbohydrate metabolism”, “energy metabolism”, and “membrane transport” (Fig. 4a). While some amino acid metabolism pathways exhibited negative differential abundance scores, other pathways exhibited positive scores, indicating a prevalence of upregulated metabolites within these pathways (Fig. 5a). These findings corresponded to the increased amino acid and carbohydrate abundances (Fig. 4d). Among the essential amino acids identified in amino acid metabolism pathways, six—L-lysine, histidine, phenylalanine, DL-threonine, L-methionine, and L-leucine—exhibited higher abundance in Lac33-inoculated samples (Fig. 5b), highlighting the enhanced nutrient value of Lac33-inoculated rice straw silage for ruminants [35, 36]. Remarkably, the “ABC transporters” pathway, responsible for metabolite transport in microbes [37], displayed upregulation in Lac33-inoculated samples, suggesting an augmented supply of metabolites as vital energy sources to support other metabolic pathways.

### Interactions between specific bacteria and metabolites in rice straw silage

**Fig. 6:**
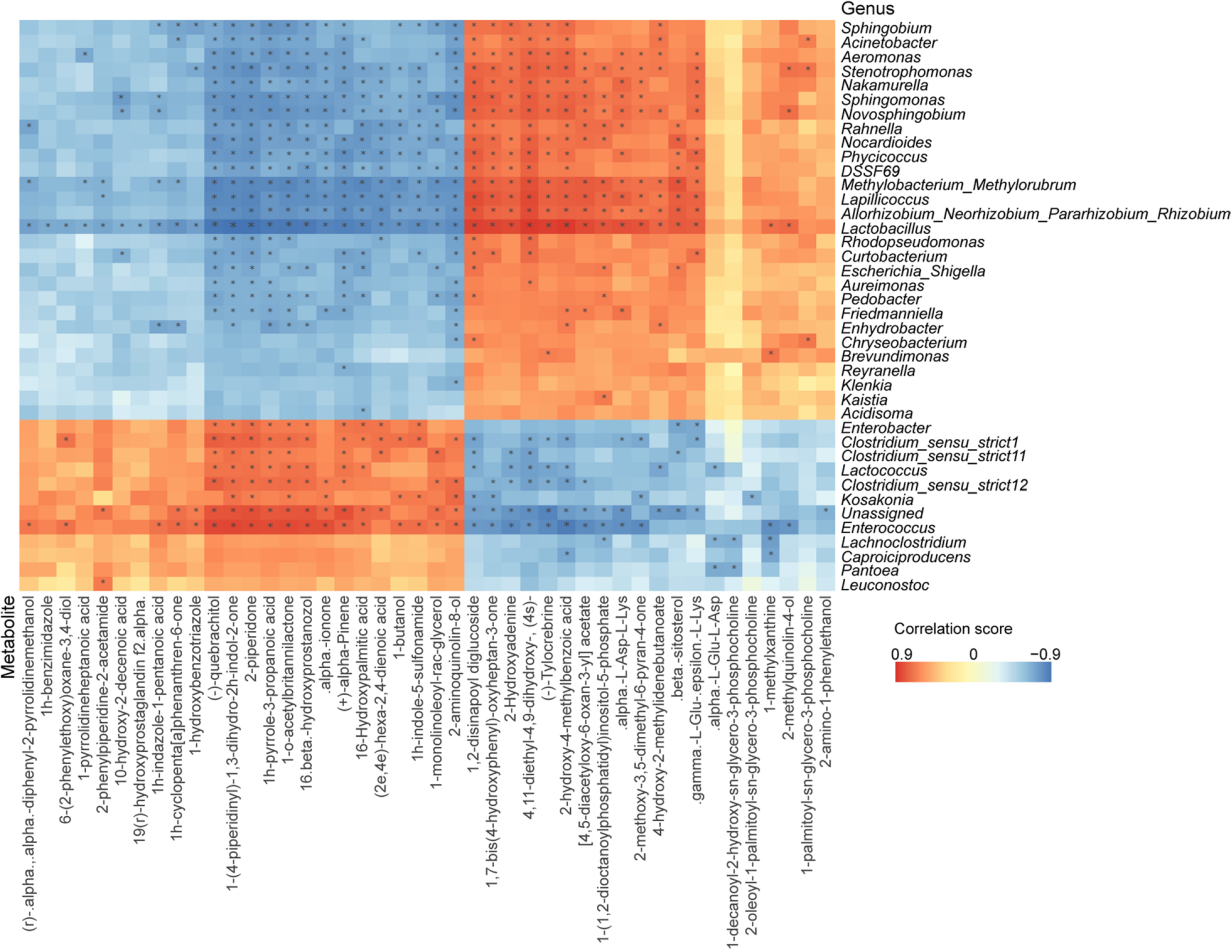
Correlations between differential bacterial genera and metabolites between silage groups with and without *L. brevis* R33 inoculation. Heatmap constructed based on Spearman’s rank correlation analysis. Asterisks denote significant correlations (Spearman’s rho > 0.6 and < -0.6, *P* < 0.05)

The intricate interplay between specific bacteria and metabolites was subsequently explored. The heatmap revealed that *Lactobacillus* exhibited the most significant correlations with identified metabolites (Fig. 6), signifying the potential influence of *Lactobacillus* dominance on the metabolite profile. Notably, amino acids alpha-L-Asp-L-Lys and gamma-L-Glu-epsilon-L-Lys demonstrated positive correlations with *Lactobacillus*. This suggests that *Lactobacillus* might stimulate the generation of beneficial peptides, thereby enhancing the nutrient value of rice straw silage [38]. Additionally, metabolites exhibiting positive correlations with *Lactobacillus* displayed negative correlations with undesired microbes like *Enterobacter* and *Clostridium*, aligning with the observed inhibition of these microbes due to *Lactobacillus* colonization (Figs. 2–3). Nevertheless, comprehensive investigations are warranted to unravel the mechanisms underlying the quality enhancement of rice straw silage through bacterial-metabolite interactions.

## Conclusions

The introduction of *L. brevis* R33 has been demonstrated to significantly enhance the quality of fermentation. This is evidenced by the reduction in NDF and ADF contents, pH, butyric acid content, and ammonia-N levels, accompanied by a concomitant increase in IVDMD, lactic acid, and propionic acid content. Through comprehensive scrutiny of bacterial communities, we unveil the dominance of *Lactobacillus* and the concurrent suppression of *Enterobacter* and *Clostridium* across various levels. Augmenting our comprehension, the metabolomic analysis illuminates the mechanisms underlying the quality enhancement of rice straw silage achieved through *L. brevis* R33 inoculation. The enrichment of carbohydrates and amino acids furnishes a potential explanation for the ascendancy of *L. brevis* R33.

## Materials and methods

### Strain isolation and identification

The *Lactobacillus buchneri* R17 strain, previously isolated from oats silage, was utilized in this study. Additionally, *L. brevis* R33 and *L. pseudomesenteroides* were isolated from oats silage using selective agar plates. To isolate these strains, an oats silage sample (0.5 g) was blended with 20 ml of distilled water. This solution was then serially diluted and cultured on selective agar plates containing sodium carboxymethyl cellulose as the sole carbon source, incubated at 28 ℃ for three days. Colonies were selected based on morphology and subjected to four consecutive replatings on MRS agar plates. Single colonies were subsequently identified through Sanger sequencing, employing the primer pairs 27F (5′-AGAGTTTGATCCTGGC. -TCAG-3′) and 1492R (5′-TACGGCTACCTTGTTACGACTT-3′). Activities of exoglucanase, endoglucanase, and β-glucosidase were assessed using methods outlined by Meng et al. [39].

### Raw materials and silage preparation

Overnight cultures of the three LAB strains were adjusted to a final concentration of OD600 0.5, generating nine inoculants: (1) CK-distilled water; (2) LB, LB medium and distilled water; (3) Leu, *L. pseudomesenteroides* culture; (4) Lac17, *L. buchneri* R17 culture; (5) Leu + Lac17, mixture of *L. pseudomesenteroides* and *L. buchneri* R17 cultures; (6) Lac33, *L. brevis* R33 culture; (7) Leu + Lac33, mixture of *L. pseudomesenteroides* and *L. brevis* R33 cultures; (8) Lac17 + 33, mixture of *L. buchneri* R17 and *L. brevis* R33 cultures; (9) Leu + Lac17 + 33, mixture of *L. pseudomesenteroides*, *L. buchneri* R17, and *L. brevis* R33 cultures. Rice (*Oryza sativa* L.) straw was chopped into 1–2 cm pieces, and 50 g of air-dried rice straws (NDF 72.18%, ADF 46.99%) were thoroughly mixed with the aforementioned inoculants to achieve a final concentration of 10^6^ CFU/g. This mixed straw (moisture content ~ 65%) was placed within a silo bag, sealed with a vacuum extractor (Dafeng, Wenzhou, China), and prepared in six replicates per treatment. Bags were stored at an ambient temperature (25–26 ℃) for 14 days, after which the samples were used for subsequent analyses.

### Assessment of chemical composition of silage and quality of fermentation

The dry matter content of rice straw silage was determined by drying at 105 ℃ to constant weight. Dry matter loss (DML) was calculated using the formula: DML (%) = (Dry Matter weight of rice straw pre-ensiled—Dry Matter weight of rice straw post-ensiled)/Dry Matter weight of rice straw pre-ensiled × 100. To evaluate fermentation quality, 5 g of silage sample was blended with 45 ml of double distilled water. The resulting slurry was filtered using five layers of medical gauze to obtain the extract. Extract pH was measured using a PHSJ-4F pH meter (Leici, Shanghai, China). The organic acids (lactic acid, acetic acid, propionic acid, and butyric acid) were quantified based on the methods described by Li et al. [33]. NDF, ADF, and acid detergent lignin (ADL) contents were determined using the methods outlined by Van Soest et al. [40] and Goering and Van Soest [41]. Total carbon (TC) and total nitrogen (TN) were assessed using an automatic elemental analyzer Vario Macro Cube (Elementar, Langenselbold, Germany). Ammonia nitrogen (NH_4_^+^-N) was determined according to Broderick and Kang [42], and NH_4_^+^-N/TN ratios were subsequently calculated. In vitro dry matter digestibility (IVDMD) of rice straw silage was determined over 12 h, 24 h, 48 h, and 72 h, utilizing the method by Tang et al. [43].

### Bacterial community analysis of rice straw silage

Genomic DNA of microbial communities present in 2 g of frozen silage sample was extracted using the E.Z.N.A. stool DNA Kit (Omega Biotek, Norcross, GA, USA). Concentrations and qualities of extracted DNA were quantified using the NanoDrop 2000 spectrophotometer (Thermo Fisher Scientific, Waltham, MA, USA). The V3-V4 variable region of the bacterial 16S rRNA gene was targeted using the universal primer pair 341F (5’-CCTACGGGNGGCWGCAG-3’) and 806R (5’-GGACTACHVGGGTWTCTAAT-3’), resulting in individual barcoded libraries. Libraries from different treatments were pooled and subjected to 250-bp pair-end sequencing using the Illumina NovaSeq 6000 platform (Illumina Inc., San Diego, CA, USA). Raw pair-end reads were analyzed using the Qiime2 platform (https://qiime2.org/). Amplicon sequence variants (ASVs) were obtained by eliminating low-quality data using DADA2 [44]. Subsequently, ASVs were taxonomically annotated against the SILVA database (https://www.arb-silva.de/, Release 138) using mothur [45].

### Metabolomic analysis of rice straw silage

To delve into the metabolic changes of rice straw silage, the frozen silage samples (*n* = 6) were thawed at 4 °C and then mixed with a pre-cooled solution of acetonitrile, methanol, and water (2:2:1, *v*/*v*) for metabolite extraction. The mixture was then centrifuged at 14000 g for 20 min at 4 °C. The resulting supernatant underwent freeze-drying and were reconstituted in a 100 μL of solution of acetonitrile and water (1:1, v/v) for further analysis. Metabolite analysis was carried out using the Agilent 1290 Infinity LC UHPLC system (Agilent Technologies, Santa Clara, CA, USA) with a ACQUIY UPLC BEH Amide column (2.1 × 100 mm, 1.7 µm, Waters, Milford, MA, USA) in combination with the AB Triple TOF 6600 Mass Spectrometer (AB SCIEX, Boston, MA, USA). The mobile phase for UHPLC consisted of solvent A: 25 mM ammonium hydroxide and 25 mM ammonium acetate in water, and solvent B: 100% acetonitrile. Then, a 12-min gradient was created as follows: *t* = 0–0.5 min, 95% solvent B; *t* = 0.5–7 min, 95% solvent B was linearly reduced to 65%; *t* = 7–8 min, 65% solvent B was linearly reduced to 40%; *t* = 8–9 min, 40% solvent B; *t* = 9–9.1 min, 40% solvent B was linearly increased to 95%; *t* = 9.1–12 min, 95% solvent B. In addition, the column temperature was 25 ℃, the flow rate was 500 μL/min, and the inject volume was 2 μL. The metabolites were then identified using positive and negative ions electrospray ionization (ESI). The ESI source conditions were set as follows: Ion Source Gas1: 60, Ion Source Gas2: 60, curtain gas: 30 psi, source temperature: 600 ℃, and IonSpray Voltage Floating: ± 5500 V. In MS only acquisition, the *m/z* range was 60–1000 Da, and the accumulation time was 0.20 s/spectra for TOF MS scan. In auto MS/MS acquisition, the *m/z* range was 25–1000 Da, and the accumulation time was 0.05 s/spectra for product ion scan, which was performed using information dependent acquisition (IDA) with high sensitivity mode. Raw data were initially converted to mzXML format using ProteoWizard (https://github.com/ProteoWizard) and subsequently handled with peak alignment, retention time adjustment, and extraction of the peak area using the XCMS software (https://github.com/sneumann/xcms). The parameters for peak picking were set as follows: centWave *m/z* = 10 ppm, peakwidth = c (10, 60), prefilter = c (10, 100). The parameters for peak grouping were set as follows: bw = 5, mzwid = 0.025, minfrac = 0.5. The annotation of isotopes and adducts was performed using CAMERA (Collection of Algorithms of MEtabolite pRofile Annotation) [46]. For the extracted ion features, only the features with more than half of the non-zero values in at least one group were retained. Compound identification of metabolites was then accomplished by comparing the accurate *m/z* values (< 10 ppm) with an in-house database (Shanghai Applied Protein Technology Co., Ltd) comprising available authentic standards [47]. In addition, the retention time, molecular mass of metabolites (mass error < 25 ppm), secondary fragmentation spectra, and also the collision energy were matched against the local database, and the identification level was higher than level 3 of the metabolite identification confidence that proposed by Schrimpe-Rutledge et al. [48].

### Data analysis and statistical analysis

Statistical analysis was conducted using Tukey's honestly significant difference test, with a significance level of *P* < 0.05. To explore the variation in bacterial communities, Principal Coordinates Analysis (PCoA) was performed using the "vegan" package in R v4.2.3. Additionally, Principal Component Analysis (PCA) of metabolites was executed using the same package, and permutational multivariate analysis of variance (PERMANOVA) was employed to assess the statistical significance of ordination plots. The Shannon indices for different bacterial communities were computed using the "vegan" package in R v4.2.3. Enrichment and depletion analyses of Amplicon Sequence Variants (ASVs) and regulation analyses of metabolites between groups were executed using the Manhattan plot (via the "CMplot" package) and Volcano plot (via the "ggVolcano" package), respectively, in R v4.2.3. The KEGG pathway enrichment analysis of differential metabolites was conducted using the "tidyverse," "magrittr," and "clusterProfiler" packages in R v4.2.3. Furthermore, the correlation between differential bacterial genera and differential metabolites was explored through pairwise Spearman's rank correlation analysis, utilizing the "psych" package in R v4.2.3.

### Supplementary Information

**Additional file 1: Figure S1.** Crude protein content of rice straw silage with inoculation of different strains. **Figure S2.** Enzyme activities of *L. brevis* R33. **Figure S3.** Abundance of ferulic acid in the silage groups with and without inoculation of *L. brevis* R33. **Table S1.** Statistical significance of the clustering pattern in ordination plots of the bacterial communities of rice straw silage with inoculations of different strains. **Table S2.** Statistical significance of the clustering pattern in ordination plots of the metabolite profiles of rice straw silage with inoculations of different strains.

**Additional file 2.** All identified metabolites of rice straw silage with inoculations of different strains.

## Data Availability

All data generated and analyzed during this study were included in this Manuscript. The raw data of high-throughput sequencing will be provided upon request.
