# Supplementary material for: Integrated microbiology and metabolomic analysis reveal the improvement of rice straw silage quality by inoculation of Lactobacillus brevis
Source: Biotechnol Biofuels Bioprod. 2023 Nov 28;16:184. doi: 10.1186/s13068-023-02431-y (PMC10685638; doi:10.1186/s13068-023-02431-y)
Supplement: Supplementary file 1 — Additional file 1: Figure S1. Crude protein content of rice straw silage with inoculation of different strains. Figure S2. Enzyme activities of L. brevis R33. Figure S3. Abundance of ferulic acid in the silage groups with and without inoculation of L. brevis R33. Table S1. Statistical significance of the clustering pattern in ordination plots of the bacterial communities of rice straw silage with inoculations of different strains. Table S2. Statistical significance of the clustering pattern in ordination plots of the metabolite profiles of rice straw silage with inoculations of different strains. [file 13068_2023_2431_MOESM1_ESM.docx]

**Integrated microbiology and metabolomic analysis reveal the improvement of rice straw silage quality by inoculation of *Lactobacillus brevis***

Yu Sun^a, b^, Qinglong Sun^c, d^, Qingyang Li^a, c^, Yunmeng Tang^a, c^, Chunjie Tian^a, b*^, Haixia Sun^a, c*^

^a^ State Key Laboratory of Black Soils Conservation and Utilization, Northeast Institute of Geography and Agroecology, Chinese Academy of Sciences, Changchun 130102, China;

^b^ Key Laboratory of Mollisols Agroecology, Northeast Institute of Geography and Agroecology, Chinese Academy of Sciences, Changchun 130102, China;

^c^ Northeast Institute of Geography and Agroecology, Chinese Academy of Sciences, Harbin 150006, China;

^d^ Northeast Agricultural University, Harbin 150006, China.

***Corresponding authors**

Chunjie Tian: E-mail, tiancj@iga.ac.cn; State Key Laboratory of Black Soils Conservation and Utilization, Northeast Institute of Geography and Agroecology, Chinese Academy of Sciences, Changchun 130102, China;

Haixia Sun: E-mail, sunhx@iga.ac.cn; State Key Laboratory of Black Soils Conservation and Utilization, Northeast Institute of Geography and Agroecology, Chinese Academy of Sciences, Harbin 150081, China.


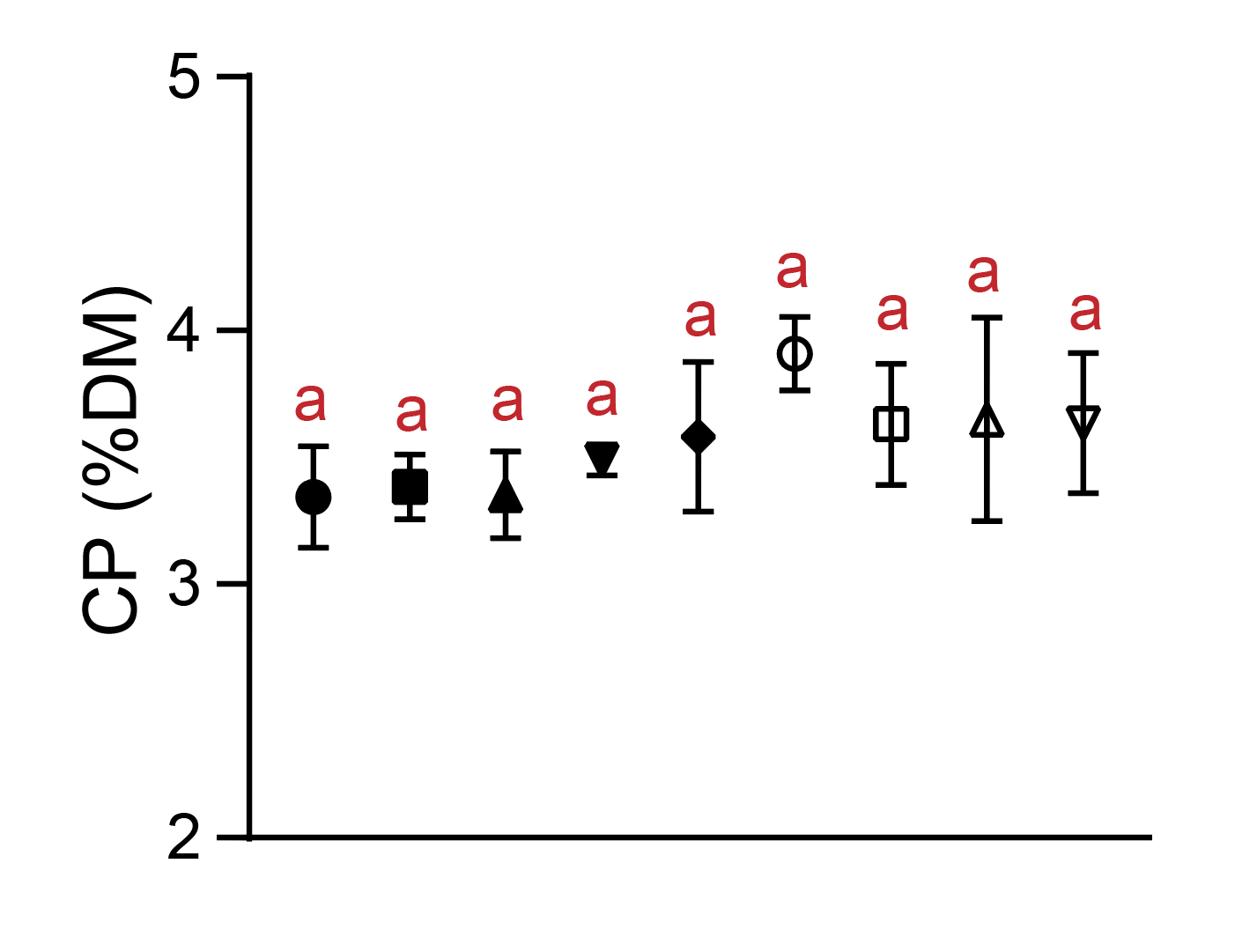


**Figure S1. Crude protein content of rice straw silage with inoculation of different strains.**

Symbols and error bars indicate the mean value and standard deviation of six biological replicates, respectively. Bars with different letter on top have significant differences in all combinations (Tukey’s honestly significant difference test, *P* < 0.05). Dashed rectangles in red and blue represent the groups that inoculated without Lac33 and with Lac33, respectively. CK, no inoculation; LB, inoculated with LB; Leu, inoculated with *Leuconostoc pseudomesenteroides*; Lac17, inoculated with *Lactobacillus buchneri* R17; Leu+Lac17, inoculated with *L. pseudomesenteroides* and *L.* *buchneri* R17; Lac33, inoculated with *L. brevis* R33; Leu+Lac33, inoculated with *L. pseudomesenteroides* and *L.* *brevis* R33; Lac17+33, inoculated with *L. buchneri* R17 and *L.* *brevis* R33; Leu+Lac17+33, inoculated with *L. pseudomesenteroides*, *L.* *buchneri* R17, and *L.* *brevis* R33. CP, crude protein; DM, dry matter.


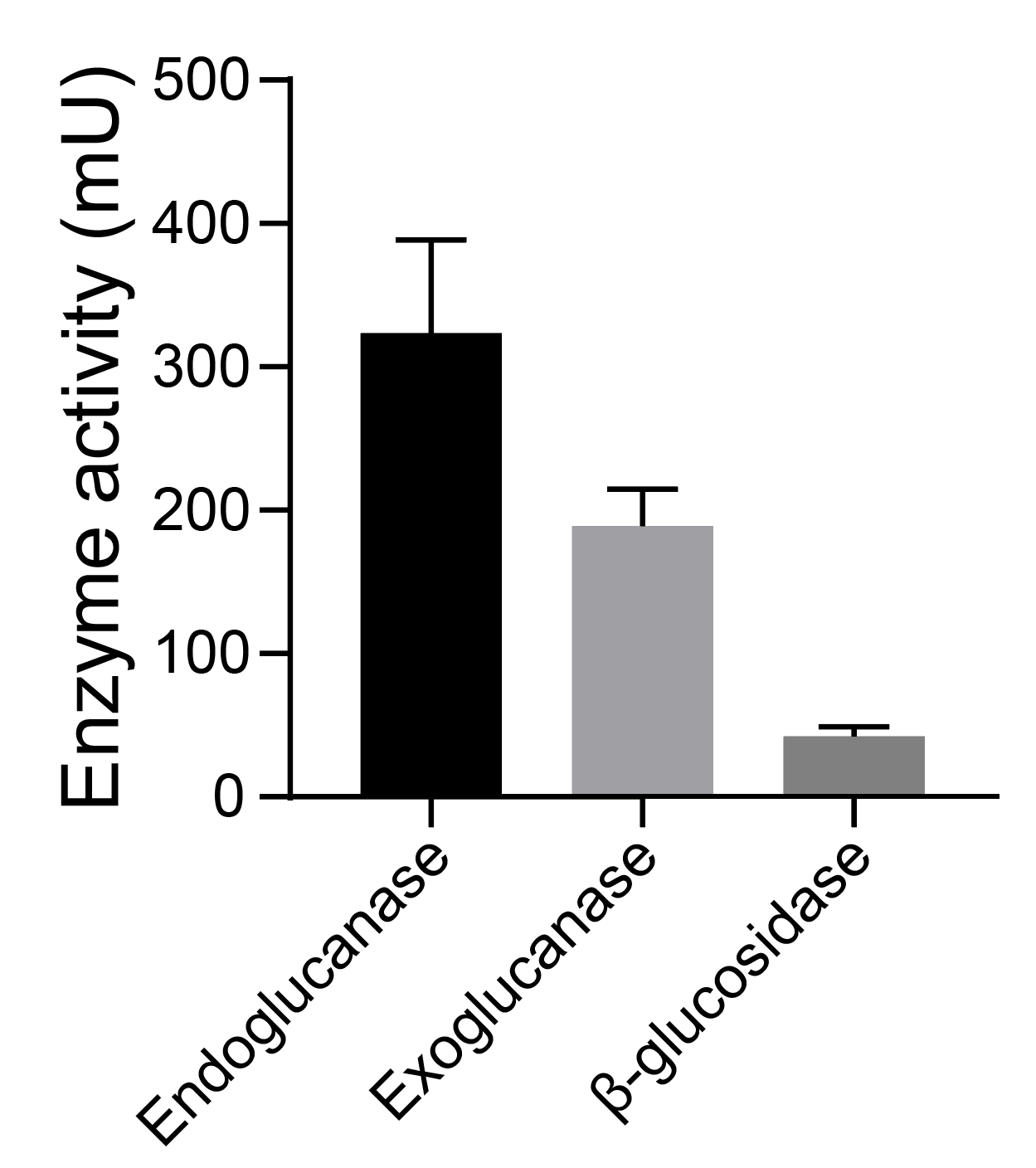


**Figure S2. Enzyme activities of *L. brevis* R33.**

Column and error bars indicate the mean value and standard deviation of biological replicates (n = 6), respectively.


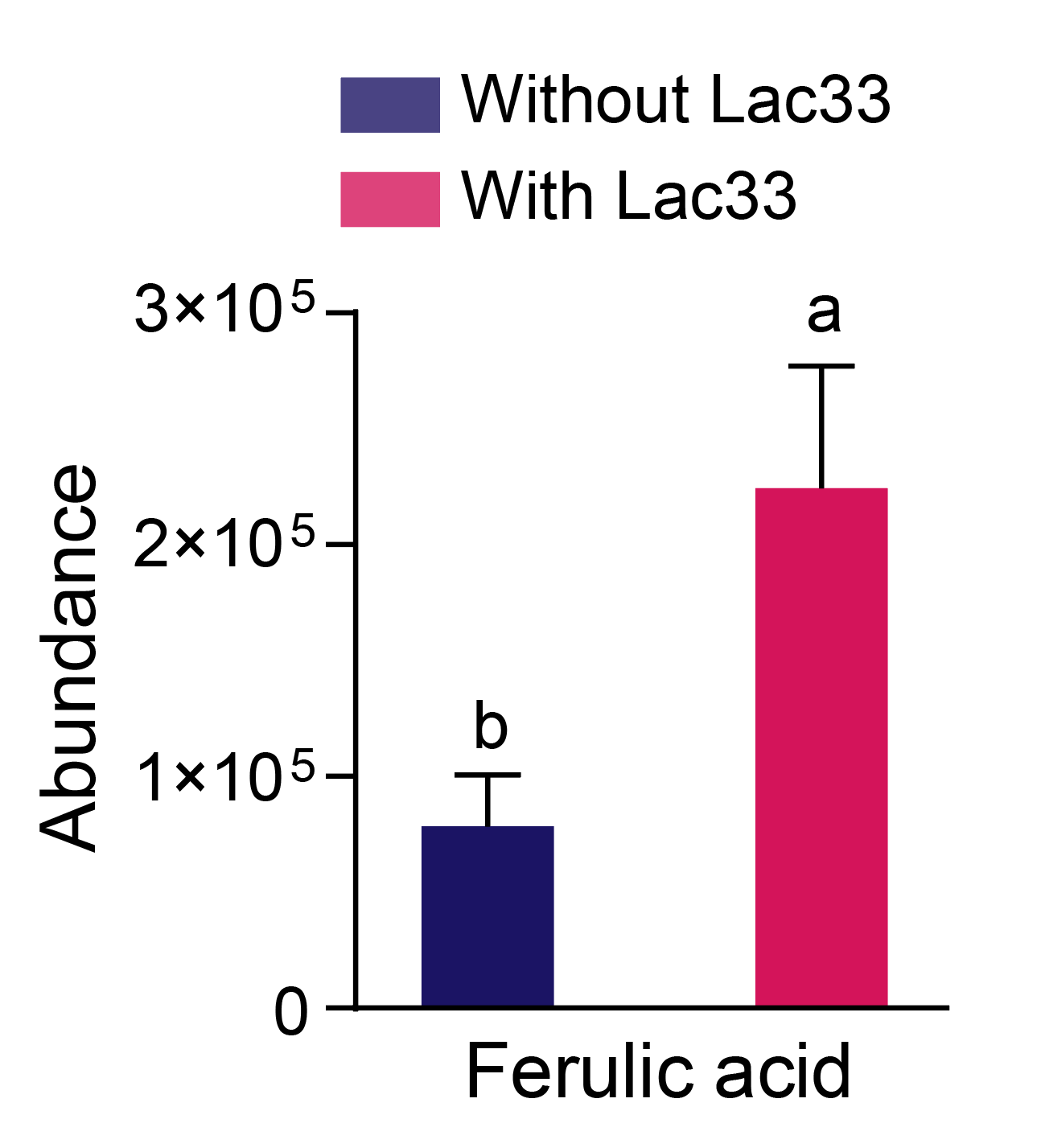


**Figure S3. Abundance of ferulic acid in the silage groups with and without inoculation of *L. brevis* R33.**

Column and error bars indicate the mean value and standard deviation of biological replicates (Without R33, n = 30; With R33, n = 24), respectively. Bars with different letter on top have significant differences (Tukey’s honestly significant difference test, *P* < 0.05).

Table S1. Statistical significance of the clustering pattern in ordination plots of the bacterial communities of rice straw silage with inoculations of different strains^a^.

| Group^a^ | *R^2^* | *p* |
| --- | --- | --- |
| With Lac33 vs. Without Lac33 | 0.74 | **< 0.001** |
| CK vs. LB | 0.17 | 0.197 |
| CK vs. Leu | 0.35 | 0.035 |
| CK vs. Lac17 | 0.44 | 0.005 |
| CK vs. Leu+Lac17 | 0.32 | 0.047 |
| CK vs. Lac33 | 0.86 | **< 0.001** |
| CK vs. Leu+Lac33 | 0.87 | **< 0.001** |
| CK vs. Lac17+33 | 0.87 | **< 0.001** |
| CK vs. Leu+Lac17+33 | 0.88 | **< 0.001** |
| LB vs. Leu | 0.25 | 0.067 |
| LB vs. Lac17 | 0.33 | 0.041 |
| LB vs. Leu+Lac17 | 0.17 | 0.197 |
| LB vs. Lac33 | 0.79 | **< 0.001** |
| LB vs. Leu+Lac33 | 0.80 | **< 0.001** |
| LB vs. Lac17+33 | 0.80 | **< 0.001** |
| LB vs. Leu+Lac17+33 | 0.81 | **< 0.001** |
| Leu vs. Lac17 | 0.44 | 0.005 |
| Leu vs. Leu+Lac17 | 0.23 | 0.071 |
| Leu vs. Lac33 | 0.84 | **< 0.001** |
| Leu vs. Leu+Lac33 | 0.86 | **< 0.001** |
| Leu vs. Lac17+33 | 0.86 | **< 0.001** |
| Leu vs. Leu+Lac17+33 | 0.87 | **< 0.001** |
| Lac17 vs. Leu+Lac17 | 0.35 | 0.035 |
| Lac17 vs. Lac33 | 0.86 | **< 0.001** |
| Lac17 vs. Leu+Lac33 | 0.87 | **< 0.001** |
| Lac17 vs. Lac17+33 | 0.87 | **< 0.001** |
| Lac17 vs. Leu+Lac17+33 | 0.88 | **< 0.001** |
| Leu+Lac17 vs. Lac33 | 0.79 | **< 0.001** |
| Leu+Lac17 vs. Leu+Lac33 | 0.81 | **< 0.001** |
| Leu+Lac17 vs. Lac17+33 | 0.81 | **< 0.001** |
| Leu+Lac17 vs. Leu+Lac17+33 | 0.82 | **< 0.001** |
| Lac33 vs. Leu+Lac33 | 0.18 | 0.198 |
| Lac33 vs. Lac17+33 | 0.20 | 0.138 |
| Lac33 vs. Leu+Lac17+33 | 0.18 | 0.198 |
| Leu+Lac33 vs. Lac17+33 | 0.12 | 0.617 |
| Leu+Lac33 vs. Leu+Lac17+33 | 0.14 | 0.317 |
| Lac17+33 vs. Leu+Lac17+33 | 0.11 | 0.700 |

^a^ CK, no inoculation; LB, inoculated with LB; Leu, inoculated with *Leuconostoc pseudomesenteroides*; Lac17, inoculated with *Lactobacillus buchneri* R17; Leu+Lac17, inoculated with *L. pseudomesenteroides* and *L.* *buchneri* R17; Lac33, inoculated with *L. brevis* R33; Leu+Lac33, inoculated with *L. pseudomesenteroides* and *L.* *brevis* R33; Lac17+33, inoculated with *L. buchneri* R17 and *L.* *brevis* R33; Leu+Lac17+33, inoculated with *L. pseudomesenteroides*, *L.* *buchneri* R17, and *L.* *brevis* R33. The statistical significance of the clustering pattern in ordination plots were determined using permutational multivariate analysis of variance (PERMANOVA).

Table S2. Statistical significance of the clustering pattern in ordination plots of the metabolite profiles of rice straw silage with inoculations of different strains^a^.

| Group^a^ | *R^2^* | *p* |
| --- | --- | --- |
| With Lac33 vs. Without Lac33 | 0.53 | **< 0.001** |
| CK vs. LB | 0.13 | 0.051 |
| CK vs. Leu | 0.15 | 0.042 |
| CK vs. Lac17 | 0.19 | 0.023 |
| CK vs. Leu+Lac17 | 0.19 | 0.023 |
| CK vs. Lac33 | 0.63 | **< 0.001** |
| CK vs. Leu+Lac33 | 0.62 | **< 0.001** |
| CK vs. Lac17+33 | 0.66 | **< 0.001** |
| CK vs. Leu+Lac17+33 | 0.66 | **< 0.001** |
| LB vs. Leu | 0.13 | 0.051 |
| LB vs. Lac17 | 0.16 | 0.023 |
| LB vs. Leu+Lac17 | 0.15 | 0.042 |
| LB vs. Lac33 | 0.60 | **< 0.001** |
| LB vs. Leu+Lac33 | 0.59 | **< 0.001** |
| LB vs. Lac17+33 | 0.63 | **< 0.001** |
| LB vs. Leu+Lac17+33 | 0.63 | **< 0.001** |
| Leu vs. Lac17 | 0.15 | 0.042 |
| Leu vs. Leu+Lac17 | 0.13 | 0.051 |
| Leu vs. Lac33 | 0.61 | **< 0.001** |
| Leu vs. Leu+Lac33 | 0.59 | **< 0.001** |
| Leu vs. Lac17+33 | 0.64 | **< 0.001** |
| Leu vs. Leu+Lac17+33 | 0.64 | **< 0.001** |
| Lac17 vs. Leu+Lac17 | 0.12 | 0.071 |
| Lac17 vs. Lac33 | 0.57 | **< 0.001** |
| Lac17 vs. Leu+Lac33 | 0.55 | **< 0.001** |
| Lac17 vs. Lac17+33 | 0.60 | **< 0.001** |
| Lac17 vs. Leu+Lac17+33 | 0.60 | **< 0.001** |
| Leu+Lac17 vs. Lac33 | 0.59 | **< 0.001** |
| Leu+Lac17 vs. Leu+Lac33 | 0.58 | **< 0.001** |
| Leu+Lac17 vs. Lac17+33 | 0.62 | **< 0.001** |
| Leu+Lac17 vs. Leu+Lac17+33 | 0.62 | **< 0.001** |
| Lac33 vs. Leu+Lac33 | 0.13 | 0.051 |
| Lac33 vs. Lac17+33 | 0.17 | 0.021 |
| Lac33 vs. Leu+Lac17+33 | 0.16 | 0.023 |
| Leu+Lac33 vs. Lac17+33 | 0.18 | 0.018 |
| Leu+Lac33 vs. Leu+Lac17+33 | 0.17 | 0.021 |
| Lac17+33 vs. Leu+Lac17+33 | 0.14 | 0.065 |

^a^ CK, no inoculation; LB, inoculated with LB; Leu, inoculated with *Leuconostoc pseudomesenteroides*; Lac17, inoculated with *Lactobacillus buchneri* R17; Leu+Lac17, inoculated with *L. pseudomesenteroides* and *L.* *buchneri* R17; Lac33, inoculated with *L. brevis* R33; Leu+Lac33, inoculated with *L. pseudomesenteroides* and *L.* *brevis* R33; Lac17+33, inoculated with *L. buchneri* R17 and *L.* *brevis* R33; Leu+Lac17+33, inoculated with *L. pseudomesenteroides*, *L.* *buchneri* R17, and *L.* *brevis* R33. The statistical significance of the clustering pattern in ordination plots were determined using permutational multivariate analysis of variance (PERMANOVA).
